# Differential Effects of Dry Eye Disorders on Metabolomic Profile by ^1^H Nuclear Magnetic Resonance Spectroscopy

**DOI:** 10.1155/2014/542549

**Published:** 2014-05-21

**Authors:** Carmen Galbis-Estrada, Sebastián Martinez-Castillo, José M. Morales, Bárbara Vivar-Llopis, Daniel Monleón, Manuel Díaz-Llopis, María Dolores Pinazo-Durán

**Affiliations:** ^1^Ophthalmic Research Unit “Santiago Grisolía”, University Hospital Doctor Peset, Avenue Gaspar Aguilar 90, 46017 Valencia, Valencia, Spain; ^2^Ophthalmic Research Unit, Faculty of Medicine, University of Valencia, Valencia, Spain; ^3^University and Polytechnic Hospital La Fe, Valencia, Spain; ^4^Central Unit of Research in Medicine, University of Valencia, Valencia, Spain; ^5^Clinical Hospital Research Foundation (INCLIVA), Valencia, Spain

## Abstract

We used ^1^H NMR spectroscopy to analyze the metabolomic profile of reflex tears from patients with dry eye disorders (DEDs). 90 subjects were divided into 2 groups: (1) patients with DEDs (DEDG; *n* = 55) and (2) healthy subjects (CG; *n* = 35). Additionally, the DEDG was subdivided into 2 subgroups based on DED severity: mild-to-moderate and moderate (*n* = 22 and *n* = 33, resp.). Personal interviews and systematized ophthalmologic examinations were carried out. Reflex tears (20–30 **μ**L) were collected by gently rubbing in the inferior meniscus of both eyelids with a microglass pipette and stored at −80°C until analysis. NMR spectra were acquired using a standard one-dimensional pulse sequence with water suppression. Data were processed and transferred to MATLAB for further chemometric analysis. Main differences in tear composition between DEDG and CG were found in cholesterol, *N*-acetylglucosamine, glutamate, creatine, amino-*n*-butyrate, choline, acetylcholine, arginine, phosphoethanolamine, glucose, and phenylalanine levels. This metabolic fingerprint helped also to discriminate between the three additional subgroups of DEDG. Our results suggest that tear metabolic differences between DEDG and CG identified by NMR could be useful in understanding ocular surface pathogenesis and improving biotherapy.

## 1. Introduction

The ocular surface (OS) is a functional unit consisting of the eyelids, lacrimal glands, cornea, conjunctiva, and tear film. Main roles of the OS are to preserve the anatomical and physiological properties of its components, to maintain the optic conditions of the eye surface dioptric, and to protect the eyes against injury by exogenous and endogenous agents [1–3]. A healthy tear film is fundamental for supporting OS integrity and refractive functions [1].

Dry eye disorders (DEDs) are complex pathological conditions involving the OS [2, 3]. Approximately 20–50% of the patients who visit an ophthalmology clinic complain of dryness, making DEDs one of the most frequent ocular morbidities. In fact, DEDs are a growing public health problem worldwide [3]. DEDs usually affect the elderly and postmenopausal women [4, 5]. However, the prevalence of DEDs may be higher than suspected, because some patients may not report their eye problems to ophthalmologists and thus remain undiagnosed. The main symptoms of DEDs include dryness, itchiness, burning, stinging, grittiness, foreign body sensation, tearing, tired eyes, redness, and blurred vision [2–4]. Among the recognized risk factors for DEDs are aging, being female, smoking, the use of topical or systemic medications, contact lenses wearing, laser excimer refractive surgery, weather conditions, environmental pollutants, air conditioning, hormone disorders, immune system diseases, and the use of video display terminals [6]. There are two major clinical forms of DEDs: the deficient aqueous tear production type (due to lacrimal gland dysfunction) and the increased evaporative loss type (due to meibomian gland disorder), but combinations of the two forms are usually seen in clinical practice [2, 3, 7, 8]. DEDs are also classified according to severity, ranging from mild to moderate to severe forms. Significant reduction in body water content associated with aging may play a pivotal role in DEDs. Meibomian gland dropout, a reduction in the number of goblet cells, and laxity of the eyelids may also be contributing factors [7, 8].

According to a report from the 2007 International Dry Eye Workshop [2], the main pathogenic mechanisms of DEDs are tear film instability and hyperosmolarity. Oxidative stress, inflammation, and apoptosis must also be considered in this process. All conditions lead to a chain of events that induce a series of OS clinical manifestations that taken together are referred to as DEDs.

Recent reports have proposed certain molecules and genes, as well as various clinical parameters, as presumptive DED biomarkers [7, 8], including oxidant and antioxidant activities, apoptotic mediators, antibodies, cytokines and chemokines, and hormone tear levels. however, lack of specificity of these biomarkers justifies the need for a further research on DED pathogenesis.

The relevance of the metabolome—the complex array of small-molecule metabolites and metabolic by-products, including carbohydrates, peptides, and lipids, present in cells, tissues, organs, and body fluids as a result of the expression and activity of genes and proteins [9–14] to both disease and health—has long been recognized. The study of the metabolome, metabolomics, may help to establish a link between the components of the metabolome and corresponding cellular responses [15, 16]. During the past 30 years, metabolomics has been used in clinical and animal studies of several diseases, including ocular pathologies. Young and Wallace reviewed the metabolic consequences of ocular diseases and explained why the multiplexed analysis inherent to metabolomics can be expected to provide data that are uniquely useful for the assessment of ocular diseases [17].

Metabolomics is defined as “the quantitative measurement of the metabolic response of living systems to pathophysiological stimuli or genetic modification” [18, 19] and is based on analytical platforms such as proton nuclear magnetic resonance spectroscopy (^1^H-NMR) and mass spectrometry (MS), particularly, gas chromatography (GC) and liquid chromatography- (LC-) MS [18, 19]. The metabolites and their concentrations provided a dataset on which multivariate statistical analysis was performed.

The NMR technique is based on the measurements of the magnetic properties of certain atomic nuclei, for example, ^1^H, ^31^P, and ^13^C, in the metabolites. Each chemical group (CH, CH_2_, CH_3_, etc.) of each metabolite will have a unique chemical shift (in ppm). The metabolite peak will have an integral value that is directly proportional to the metabolite concentration in the sample. In MS experiments compounds are ionized to form positively or negatively charged molecules which are separated and detected according to their mass-to-charge ratio.

^1^H NMR spectroscopy analysis of biofluids provides information on both the structure and the composition of low-molecular-mass metabolites in biological fluids and is a rapid and low-cost technique for exploring pathological metabolic processes. The major advantages of NMR spectroscopy include its unbiased metabolite detection, quantitative nature, and high reproducibility [18]. Unfortunately, this technique is associated with a low sensitivity compared to the other analytical methods such as GC-MS or LC-MS. The MS analysis requires more labor-intense (and destructive) tissue preparation. Detectable compounds are limited to those that can be derivatized, which can be time consuming, costly, and carrying a risk of metabolite loss. NMR-based metabolomics could be appropriate as a cost effective solution for high-throughput analysis [20].

Biotechnological advances, such as the application of NMR spectroscopy to tissue and biofluid samples, have permitted the generation of data on low-molecular-weight metabolites in samples from individuals [9–11]. For example, Holmes et al. analyzed the aqueous humor and vitreous body of pig eyes by means of ^31^P NMR [15]. Midelfart et al. analyzed extracts of rabbit corneas and lenses by NMR spectroscopy and observed that some metabolites suggested the existence of a powerful antioxidant environment in the ocular tissues [21–23]. Our group reported previous experience on the metabolomics study by NMR of the aqueous humor in a rat model of hyaluronic acid-induced ocular hypertension [24]. Compounds identified in these glaucomatous samples correlated well with data obtained in similar glaucoma models by means of conventional techniques. Moreover, Mayordomo-Febrer et al. published a detailed work on the characterization of human tear metabolome using LC-MS/MS [25]. Although NMR study of tear composition remains incomplete, some works using this biofluid [23, 24, 26–28] have demonstrated that metabolomics can be useful for monitoring eye diseases such as DEDs.

Proteomic assays have recently been reported in tear samples of dry eye patients by 2D electrophoresis (2DE) and differential gel electrophoresis (DIGE). Presumptive biomarkers of DEDs included proline rich 4 protein (LPRR4) which appeared downregulated in all types of DEDs [29]. Other authors have suggested the higher expression of apoptotic and inflammation proteins in tears from diabetic dry eye patients as performed by two-dimensional nanoliquid chromatography coupled with tandem mass spectrometry- (MS-) based proteomics [30].

In the present study, our goal was to improve knowledge on human tear composition by using ^1^H NMR spectroscopy-based metabolite profiling, followed by multivariate statistical analysis, in order to explore metabolite imbalances between DEDG. In addition, we investigated the possibility to build up a metabolic discriminating model that helps us to identify the three established severity ranges subgroups of DED, with the ultimate goal of improving the management of these patients.

## 2. Material and Methods

All participants firmed the informed consent and all procedures of this prospective study were subjected to the Declaration of Helsinki for the protection of human subjects in medical research. The study was approved by the Institutional Review Board of the University and Polytechnic Hospital La Fe (Valencia, Spain) (Ref. 2013/0417).

### 2.1. Patients and Groups

A total of 90 subjects of both sexes, aged 25–80 years, were enrolled during ophthalmologic appointments at the study center (University and Polytechnic Hospital La Fe, Valencia, Spain) between February 2013 and September 2013 according to the main inclusion and exclusion criteria listed in Table 1.

Suitable subjects were assigned to one of the following groups: (1) patients diagnosed with DEDs (DEDG; *n* = 55) and (2) healthy subjects as a control group (CG; *n* = 35). From the DEDG, three subgroups were formed on the basis of clinical OS data and OSDI questionnaire: patients diagnosed with mild-to-moderate (*n* = 22) and moderate (*n* = 33) DEDs. Participants pertaining to the severe forms were excluded of the present study.

### 2.2. Clinical Assessment and Tear Sampling

Each subject was interviewed about their personal and familial background and their personal characteristics and lifestyle as well as disease data (symptoms of dry eyes and subjective sensations, duration, and treatments). We used the Ocular Surface Disease Index (OSDI) questionnaire as part of the interview. This clinically validated questionnaire was designed to evaluate DEDs in terms of ocular symptoms, visual function, and environmental factors. Each of the 12 questions has to be responded to with a rating from (0 to 4), and a final score (0–100) is then calculated, with higher scores indicating more-severe DEDs [24].

Each subject was given a systematized ophthalmologic examination consisting of the following tests: measurement of best corrected visual acuity in each eye, Schirmer's test, slit-lamp examination of the eye adnexa and anterior segment, and measurement of tear break-up time (BUT) with fluorescein.

Reflex tear samples were obtained by gently rubbing the inferior meniscus of both eyelids with a micro-Pasteur pipette, without damaging the OS tissues, as previously described [25]. Tears from both eyes (total volume 20–30 *μ*L) were deposited in one labeled cryotube per subject and stored at −80°C until metabolomics analysis. Patients for whom the volume of the collected tear sample was less than 10 *μ*L were rejected; because of this, it was difficult to get a significant number of severe DEDs participants.

### 2.3. ^1^H NMR Spectroscopy

For NMR analysis, 20 *μ*L of tear was mixed with 2.5 *μ*L of 0.05 mM sodium-3-trimethylsilylpropionate-2,2,3,3-d4 (TSP) in deuterium oxide (D_2_O). A total of 20 *μ*L of the mixture from each subject was then transferred to a 1 mm high-quality NMR tube. All ^1^H NMR spectra were acquired using a standard one-dimensional pulse sequence with water suppression on a Bruker Avance 600 spectrometer operating at 600.13 MHz with a 1 mm ^1^H/^13^C/^15^N TXI probe. A total of 256 free induction decays were collected into 64 k data points with a spectral width of 14 ppm and a recycle delay of 1 s. The water signal was saturated by weak irradiation during the recycle delay. Before Fourier transformation, the free induction decay was multiplied by a 0.3 Hz exponential line broadening. Spectral chemical shift referencing on the TSP signal at 0 ppm was performed for all spectra. Spectral regions between 0.5 and 4.4 ppm and between 5.5 and 9.5 ppm were binned in segments of 0.01 ppm width (6 Hz) for multivariate analysis. Binned data were normalized to total aliphatic spectral area. We used available spectral databases and two-dimensional NMR experiments to facilitate structural identification of relevant metabolites. Spectra were processed using MestRenova 6.2 software (Mestrelab Research S.L., Santiago de Compostela, Spain) and transferred to MATLAB 7.6 (The MathWorks Inc., Natick, MA, USA) for additional processing and further analysis. Signals belonging to selected metabolites were integrated and quantified using semiautomated in-house MATLAB peak-fitting routines based on Levenberg-Marquardt optimization procedures. Resonances were assigned according to the previous literature, the Human Metabolome Database (http://www.hmdb.ca/), and characteristic cross-peak from previously mentioned 2D spectra to unequivocal assignation of the resonances.

### 2.4. Statistical Analyses

Chemometrics statistical analyses were performed using in-house MATLAB scripts and PLS Toolbox 6.7 (Eigenvector Research Inc., Wenatchee, WA, USA). Principal component analysis (PCA) and partial least squares discriminant analysis (PLS-DA) were applied to NMR spectra data matrix. PLS-DA is a classification technique that combines the properties of partial least-squares (PLS) regression with the discrimination power of discriminant analysis (DA) [20]. The main advantage of PLS-DA models is that the main sources of variability in the data are modeled by the so-called latent variables and consequently in their associated scores and loadings, allowing the visualization and understanding of different patterns and relations in the data. The PLS-DA model was tested using a leave-one-out cross-validation (CV) algorithm.

All data are expressed as mean ± standard deviation (SD). Finally, one-way analysis of variance was used for the determination of statistical significance between group means of the corresponding integrals. A difference was considered significant when *P* < 0.05.

## 3. Results

Median ages of the subjects were 52 ± 18 years old (DEDG) and 36 ± 11 years old (CG). The percentages of men and women in the two groups (DEDG and CG) were 75% and 58%, 25% and 42%, respectively.

On the basis of data collected from the personal interview and the OSDI questionnaire, the ophthalmologic examination, and the reported symptomatology and subjective sensations, patients were classified according to the degree of DED severity as follows: 42% mild-to-moderate DEDs and 58% moderate DEDs.

The Schirmer test scores were lower in patients with mild-to moderate [9.80 ± 1.71 mm] and moderate DEDs [6.13 ± 2.42 mm] than in subjects in the CG [18.88 ± 5.56 mm], reflecting the altered tear secretion in the DEDG patients. The BUT scores were lower in patients with mild-to-moderate [7.52 ± 0.87 s] and moderate [5.89 ± 0.65 s] DEDs than in subjects in the CG [7.98 ± 0.06 s], which strongly suggests that the tear film stability was altered in DEDG patients.

**Figure 1:**
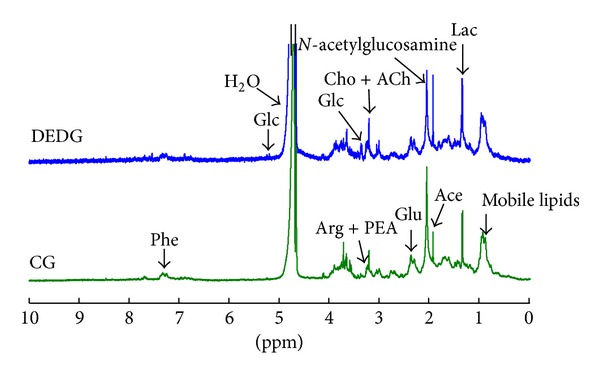
Representative spectra of tears from DEDG patients (upper spectrum) and CG subjects (lower spectrum).

Data from the ^1^H NMR spectra of the tear samples from all the study subjects permitted a visual comparison between the average spectra of tears from CG subjects and the DEDG patients. Average spectra for the two groups are shown in Figure 1 with some of the most intense metabolite peaks labeled.

**Figure 2:**
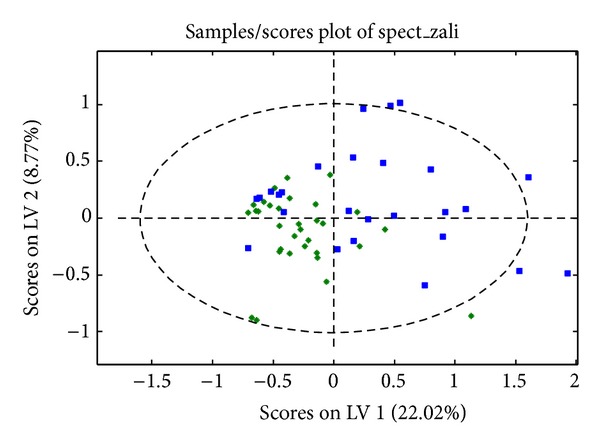
PLS-DA score plot of the DEDG (blue squares) and the CG (green asterisks).

The metabolic profiles of the groups were compared by means of principal component analysis, which is an unsupervised test for homogeneity of the set of samples (detecting the existence of possible outliers). As part of this process we determined the presence of 9 samples (3 from the CG and 6 from the DEDG) that were excluded from further analysis. Outliers are defined as those samples that are situated outside the 95% confidence interval of the Hotelling T-squared distribution in the score scatter plot. Because the samples did not cluster spontaneously, we performed a PLS-DA that maximized the separation between the groups (Figure 2). The PLS-DA score plot clearly indicated an incipient separation with minimum overlap between DEDG and the CG samples, confirming the existence of significant differences in metabolic profile between the two groups.

Quantification of the most contributing regions in the PLS-DA model allowed us to determine the major metabolites differences in the tears of the two study groups (Table 2).

**Figure 3:**
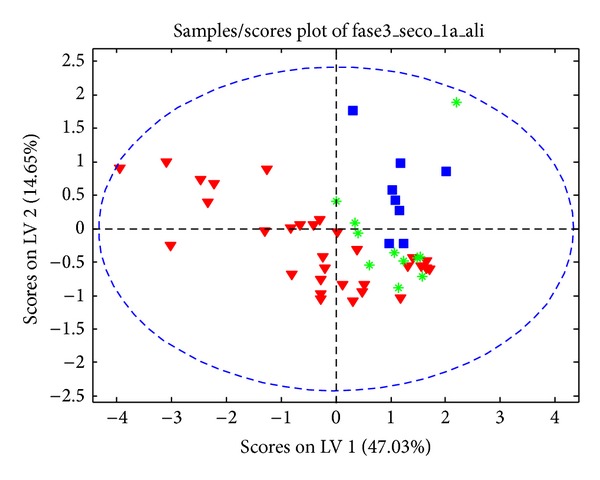
Score plot of the PLS-DA discriminatory model to differentiate between mild-to-moderate dry (red and blue) and moderate dry eyes (green).

Additionally, based on previous metabolites differences found between DEDG and CG, we proceeded with a deeper analysis in order to build up a model that helps us to discriminate between the three DED subgroups. Firstly, we constructed a PLS-DA model using the two extreme groups (severe-DED and mild-DED). After that, the intermediate group (moderate DEDs) was projected in the same discriminating space. We obtained a multivariate space where the three subgroups presented a clear differential grouping. The mild-to-moderate and the moderate DED samples were distributed as shown in Figure 3.

In Table 3 relative values of the most contributing regions in the PLS-DA discriminating model for each subgroup sample are reflected.

## 4. Discussion

The anatomy and function of the OS must be preserved by appropriate tear secretion and availability [1–4]. The tear film is formed by various layers that are secreted, in turns, by several ocular glands and tissues. DEDs are complex pathological conditions involving the OS and are among the most frequent ocular morbidities worldwide [1–8]. This research was conducted to shed light on the metabolite composition of the human tears in relation to DED manifestations and severity.

Mean age of the DED patients was 52 ± 18 years, and women made up 75% of the DEDG, in agreement with previous reports [1–5], confirming that age and sex are relevant factors in DED initiation and progression, as previously reported [5, 6]. Statistically significant differences in the Schirmer and BUT scores were observed between the two main groups, as previously reported [1–8]. We subdivided the DEDG patients according to the severity of clinical DED manifestations (mild, moderate, or severe). The results of the Schirmer and BUT tests showed different degrees of alteration that were closely related to the intensity of clinical signs and symptoms.

^1^H NMR spectroscopy allowed us to identify and quantify sets of metabolites in human tears with little sample preparation and quite small sample volumes. In addition, the metabolite profile could be acquired relatively rapidly (5 min with a short routine), with sensitivity sufficient to evaluate even subtle differences. By using the combination of NMR metabolomics and chemometric approaches, we were able to obtain and include in the analysis a large amount of data, which contribute to obtaining robust models that can be expected to provide useful information for further ophthalmic research [9–16].

The ^1^H NMR metabolic profiles of the DEDG and the CG were clearly different from each other. The major differences in tear composition, between CG and the DEDG samples, were in* N*-acetylglucosamine, glutamate, creatine, amino-*n*-butyrate, choline, acetylcholine, arginine, phosphoethanolamine, cholesterol/lipids, glucose, and phenylalanine levels. Many of these metabolites have been cited in previous works which were carried out in human tears samples, as substrate (or by-products) of the ocular surface metabolism. In Table 4 each metabolite and its bibliographical reference are summarized.

The meibomian glands secretions comprise the lipid layer of the tear film which is essential for preventing rapid evaporation of the tears [30]. The results presented herein demonstrated lower lipid levels in tears from the DEDG as compared to the controls.

The increased levels of essential amino acids such as arginine and phenylalanine in the DED reflect the proinflammatory response to this type of ocular surface alteration [31]. However it is difficult to clearly discuss the presence and function of these amino acids, due to the variability of them found in individuals with or without alteration of the ocular surface. Furthermore, oxidative stress occurring in the cellular compartments of the ocular surface, as described in subjects with DEDs, could generate increased levels of glucose and creatine in the tear film, in a similar manner as it has been described in our work [32].

There is a strong regulatory action of parasympathetic autonomous system stimulation on the secretion of lacrimal and salivary glands. Important changes in the stimulus/secretion process associated with parasympathetic stimulation have been previously described by Bacman et al. [34]. In fact, higher levels of neurotransmitters choline and acetylcholine in tears of our DEDs patients constituted an interesting finding but elucidation of their role in dry eyes is far from complete.

In an attempt to obtain additional data on OS alterations and tear composition in patients with DEDs, we discriminated the metabolome of DED tears in three subgroups of progression (mild, moderate, and severe). Our data showed that the metabolomic profiles of each one of these DEDs subgroups differed significantly. In this scenario we suggest for the first time that DED differences in severity have a significant effect in tear metabolic content, suggesting that the tear metabolomic profile may be utilized as biomarkers and surrogated endpoints of DEDs.

In summary, metabolomics is a feasible tool for measuring the metabolite profile of human tears. The results presented herein strongly suggest that further metabolomic analyses of OS pathologies should be conducted. The identification of specific metabolites associated with particular situations (oxidative stress, inflammation, and apoptosis) will enhance the usefulness of this technique in a better understanding of the pathogenic mechanisms of eye disorders and design of new therapeutic strategies [33, 35]. Finally, the combination of all this metabolomic information in a discriminating model gives us the opportunity of developing a fast, reliable, and cheap methodology for severity classification of DED patients using a small sample of tears.

## Figures and Tables

**Table 1:** Inclusion and exclusion criteria.

Inclusion criteria	Exclusion criteria
Aged 23–80 years	Aged ≤ 23 years or ≥80 years
Diagnosed with DEDs (DEDG)	Athopy, allergic disorders
Healthy subjects (CG)	Wearing contact lenses
Able to participate in the study	Not able to participate in the study
Informed consent obtained	Systemic diseases and general treatments
	Ocular disorders and use of eye drops other than artificial tears
	History of refractive surgery, ophthalmic laser treatment (≤3 months)

**Table 2:** Mean spectral intensity on the metabolites included in the discriminating model between DEDG and CG subjects.

Metabolites	Region (ppm)	*P* value	CG (au)	DEDG (au)
–CH_3_ lipids	*0.84–0.88 *	3*e* − 6	0.0264 ± 0.006	0.0202 ± 0.006
Cholesterol/lipids	*0.90–0.93 *	7*e* − 7	0.0248 ± 0.005	0.0184 ± 0.006
*N*-Acetylglucosamine	*2.0–2.08 *	8*e* − 9	0.079 ± 0.02	0.052 ± 0.02
Glutamate	*2.325–2.415 *	0.001	0.0313 ± 0.01	0.0266 ± 0.004
Total creatine	*3.02–3.05 *	9*e* − 9	0.0053 ± 9*e* − 4	0.0067 ± 0.001
Amino-*n*-butyrate	*2.95–3.025 *	0.0002	0.0135 ± 0.003	0.0157 ± 0.002
Choline/acetylcholine	*3.18–3.21 *	0.01	0.0089 ± 0.002	0.010 ± 0.002
Arginine + phosphoetanolamine	*3.21–3.28 *	3*e* − 6	0.0147 ± 0.003	0.0189 ± 0.004
Choline	*4.05–4.09 *	1*e* − 7	0.0036 ± 0.001	0.0057 ± 0.002
Glucose	*5.17–5.29 *	8*e* − 8	0.0064 ± 0.005	0.0177 ± 0.01
Phenylalanine	*7.20–7.40 *	2*e* − 7	0.0245 ± 0.005	0.0333 ± 0.008

**Table 3:** Relative concentration of tear discriminating metabolites from mild-to-moderate DEDG and moderate-DEDG patients. Values are mean ± SD. Only significant metabolites are shown (*P* < 0.05, two-sample *t*-test).

Metabolites	Region (ppm)	*P* value	Mild-to-moderate DEDG (au)	Moderate DEDG (au)
–CH_3_ lipids	*0.84–0.88 *	0.007	0.0263 ± 0.005	0.0233 ± 0.007
Cholesterol/lipids	*0.90–0.93 *	0.01	0.025 ± 0.004	0.021 ± 0.006
*N*-Acetylglucosamine	*2.0–2.08 *	0.004	0.077 ± 0.01	0.059 ± 0.02
Glutamate	*2.325–2.415 *	0.02	0.031 ± 0.002	0.027 ± 0.004
Amino-*n*-butyrate	*2.95–3.025 *	0.04	0.0141 ± 0.001	0.0142 ± 0.002
Choline	*4.05–4.09 *	0.02	0.004 ± 0.001	0.005 ± 0.002
Glucose	*5.17–5.29 *	0.007	0.0084 ± 0.005	0.015 ± 0.009
Phenylalanine	*7.20–7.40 *	0.08	0.028 ± 0.004	0.034 ± 0.006
Formate	*8.45–8.475 *	0.02	0.0018 ± 0.0008	0.003 ± 0.001

**Table 4:** Tear metabolites in the literature.

Metabolite	Reference
Cholesterol	Butovich, 2008 [26]
Glutamate	Nakatsukasa et al., 2011 [31]; Zhou and Beuerman, 2012 [33]
Arginine	Nakatsukasa et al., 2011 [31]; Zhou and Beuerman, 2012 [33]
Glucose	Taormina et al., 2007 [32]
Creatine	Zhou and Beuerman, 2012 [33]
Acetylcholine	Zhou and Beuerman, 2012 [33]
Phenylalanine	Nakatsukasa et al., 2011 [31]; Zhou and Beuerman, 2012 [33]
Choline	Zhou and Beuerman, 2012 [33]
